# U-shaped GAN for Semi-Supervised Learning and Unsupervised Domain Adaptation in High Resolution Chest Radiograph Segmentation

**DOI:** 10.3389/fmed.2021.782664

**Published:** 2022-01-13

**Authors:** Hongyu Wang, Hong Gu, Pan Qin, Jia Wang

**Affiliations:** ^1^Faculty of Electronic Information and Electrical Engineering, Dalian University of Technology, Dalian, China; ^2^Department of Surgery, The Second Hospital of Dalian Medical University, Dalian, China

**Keywords:** semi-supervised learning, unsupervised domain adaptation, generative adversarial network, medical image segmentation, chest radiograph

## Abstract

Deep learning has achieved considerable success in medical image segmentation. However, applying deep learning in clinical environments often involves two problems: (1) scarcity of annotated data as data annotation is time-consuming and (2) varying attributes of different datasets due to domain shift. To address these problems, we propose an improved generative adversarial network (GAN) segmentation model, called U-shaped GAN, for limited-annotated chest radiograph datasets. The semi-supervised learning approach and unsupervised domain adaptation (UDA) approach are modeled into a unified framework for effective segmentation. We improve GAN by replacing the traditional discriminator with a U-shaped net, which predicts each pixel a label. The proposed U-shaped net is designed with high resolution radiographs (1,024 × 1,024) for effective segmentation while taking computational burden into account. The pointwise convolution is applied to U-shaped GAN for dimensionality reduction, which decreases the number of feature maps while retaining their salient features. Moreover, we design the U-shaped net with a pretrained ResNet-50 as an encoder to reduce the computational burden of training the encoder from scratch. A semi-supervised learning approach is proposed learning from limited annotated data while exploiting additional unannotated data with a pixel-level loss. U-shaped GAN is extended to UDA by taking the source and target domain data as the annotated data and the unannotated data in the semi-supervised learning approach, respectively. Compared to the previous models dealing with the aforementioned problems separately, U-shaped GAN is compatible with varying data distributions of multiple medical centers, with efficient training and optimizing performance. U-shaped GAN can be generalized to chest radiograph segmentation for clinical deployment. We evaluate U-shaped GAN with two chest radiograph datasets. U-shaped GAN is shown to significantly outperform the state-of-the-art models.

## 1. Introduction

Recently, deep learning models have gained increasing popularity in medical segmentation. However, deep learning models with supervision require substantial pixel-level annotated data to achieve sufficient accuracy and prevent over-fitting (1–4). Pixel-level annotation is expensive, especially with medical images, because it is time-consuming and requires highly skilled experts (3, 5). Therefore, medical image datasets are usually small, which cannot meet the requirement of deep learning, due to a lack of annotations (6, 7). Even if a model is well-trained on a certain medical dataset, its accuracy decreases when it is applied to unseen domains (8, 9). The deep learning models suffer an accuracy drop between two domains due to domain shift (8). These problems limit the application of deep learning models in clinical environments.

An alternative to supervised learning is semi-supervised learning, which involves using unannotated data to identify specific hidden features of the dataset to facilitate prediction. Semi-supervised deep learning using generative adversarial networks (GANs) (10) has been highly successful (1, 2), especially with nonmedical images (3). A semi-supervised GAN model was proposed to distinguish between predicted probability maps and the ground truth (2). In Souly et al. (1), a GAN was used to generate fake images close to real images as learned by the segmentation network. In medical segmentation, a few semi-supervised models have been developed using the model in Hung et al. (2) to improve the prediction accuracy with specially designed loss functions for particular image types, such as computed tomography of the liver (3), retinal optical coherence tomography (11), and pediatric MRI (12).

Domain adaptation (DA) suffers domain shift by transferring knowledge from the source domain to the target domain (13–16). A popular solution is transfer learning, which fine-tunes parts of a pre-trained model with annotated target domain data (13). However, transfer learning approaches rely on additional annotated data on the target domain, which is expensive or sometimes impractical. Instead, unsupervised domain adaptation (UDA) is more appealing to generalize models in clinical practice. UDA using GAN is becoming increasingly popular in the medical field (14–16). GAN-based UDA models use generators to transform the target data to the source domain, discriminate the source data from the target data, and improve segmentation accuracy with a specific training method (16), net structure (14), or training loss (15).

Deep learning algorithms require large amounts of data, which cannot be collected from a single medical center. Therefore, data from multiple collection centers, comprising large medical centers and small clinics, are required (17–19). The large medical centers provide partly annotated datasets for semi-supervised learning, while the clinics provide unannotated data. The annotated and unannotated data may come from either the same or different domains in the dataset collected from multiple centers. A single model that can deal with the semi-supervised and UDA approach at the same time is urgently needed.

To tackle the aforementioned problems, we propose an improved GAN model, called U-shaped GAN, for medical image segmentation. U-shaped GAN is improved by replacing the traditional discriminator with a U-shaped net to assign each pixel a label. Training the segmentation model with images of high resolution is effective; however, it increases the computational burden (20, 21). U-shaped GAN is designed with high resolution radiographs for effective segmentation while considering computational burden. The pointwise convolution is applied to U-shaped GAN for dimensionality reduction, which decreases the number of feature maps while retaining their salient features. Moreover, the U-shaped net takes a pretrained ResNet-50 as an encoder to reduce the computational burden of training from scratch. A pixel-level semi-supervised loss is proposed to leverage the unannotated data to assist the annotated data for semi-supervised learning. U-shaped GAN is extended to UDA with minimal modification. The semi-supervised learning approach and UDA approach are merged into a single model to handle datasets from multiple medical centers conveniently and efficiently. We evaluate U-shaped GAN on lung segmentation for radiographs.

To conclude the introduction, we outline the major contributions of this work as follows:

(1) U-shaped GAN is proposed for high resolution medical image segmentation while taking computational burden into account.
(2) A semi-supervised learning approach is proposed to overcome the lack of annotated data. We employ a pixel-level semi-supervised loss that leverages the unannotated data to assist the annotated data for segmentation.
(3) U-shaped GAN is extended to UDA with minimal modification to transfer knowledge among different domains without additional annotated data on the target domain.
(4) In our framework, the semi-supervised learning approach and UDA approach are merged into a single model to handle datasets from multiple medical centers conveniently and efficiently.

## 2. Methods

### 2.1. Background

In recent years, GAN has garnered considerable attention because of its superior performance in terms of generating images (2). GAN consists of a generator network *G* and discriminator net *D*. *G* generates fake images close to real data from a noise distribution deceiving the discriminator, while *D* distinguishes the real images from fake ones. *G* and *D* can be considered as two competitors in a min-max game with the following formulation:


(1) $$\begin{array}{r} \begin{array}{r} \min_{G}\max_{D}V\left(D,G\right)=E_{x\sim p_{data}\left(x\right)}\left[\log\left(D{\left(x\right)}_{real}\right)\right]\\ \;+E_{z\sim p_{noise}\left(z\right)}\left[\log\left(1-D{\left(G\left(z\right)\right)}_{real}\right)\right], \end{array} \end{array}$$

where *E* is the expectation of a random variable, *p*_*data*_(*x*) is the real data distribution, and *p*_*noise*_(*z*) is a noise distribution. *D*(*)_*real*_ stands for the possibility that the sample is from the real data. *G* transforms the noise variable *z* from the distribution *p*_*noise*_(*z*) into *G*(*z*). The min-max game provides a useful feature representation for auxiliary supervised discrimination tasks (22).

### 2.2. Proposed Model

The goal of this study is to develop a unified framework for semi-supervised learning and UDA. Analyzing the influence factors in semi-supervised learning and UDA on chest radiographs, we propose a similar solution for the semi-supervised learning approach and UDA approach. A pixel-level semi-supervised loss is proposed to leverage the unannotated data to assist the annotated data for segmentation.

#### 2.2.1. Semi-Supervised Learning

We propose a U-shaped GAN for semi-supervised lung segmentation from chest radiograph datasets. U-shaped GAN is based on the following hypothesis: the features in an ideal representation correspond to the underlying causes of the data. If label *y* is among the salient causes of data *x*, a suitable representation for the probability distribution *p*(*x*) may also be a suitable representation for computing the distribution of conditional probability *p*(*y*|*x*) (23). The marginal probability *p*(*x*) is related to the conditional probability *p*(*y*|*x*) through the Bayes rule:


(2) $$\begin{array}{l} p\left(y|x\right)=\frac{p\left(x|y\right)p\left(y\right)}{p\left(x\right)} \end{array}$$

**Figure 1:**
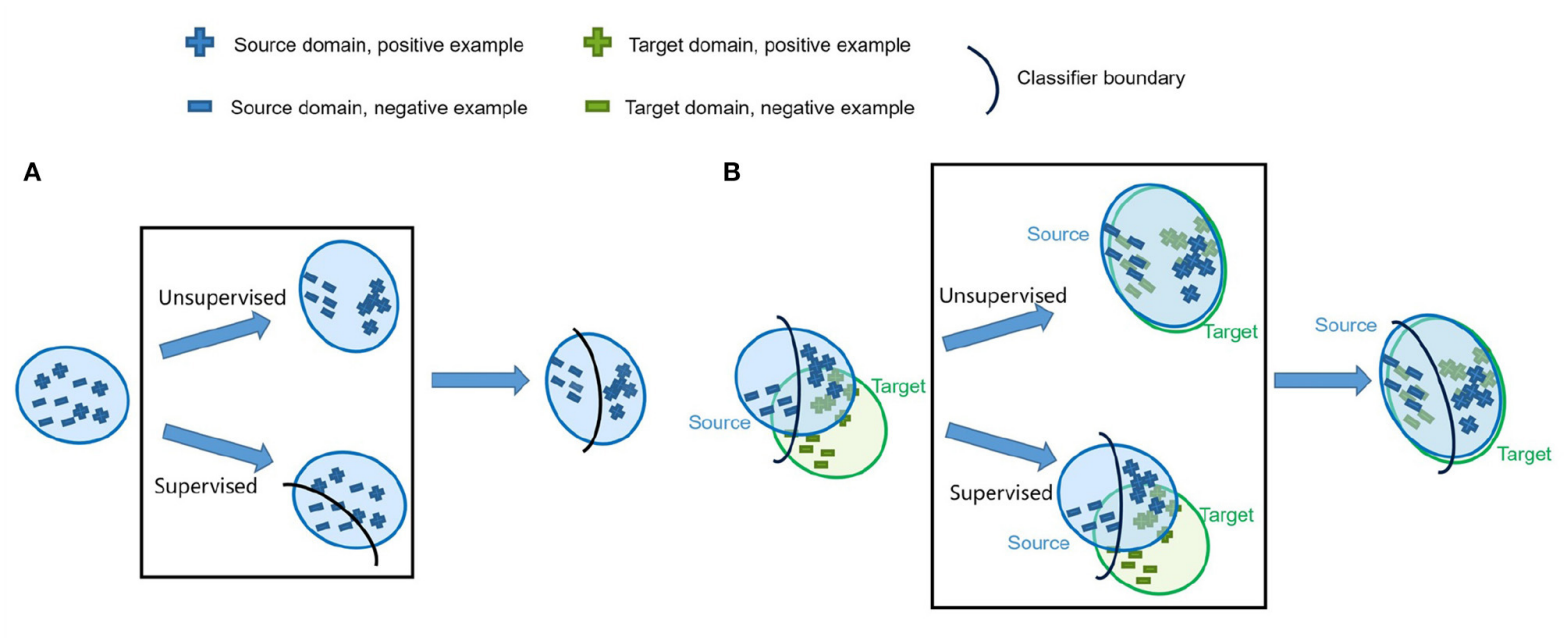
**(A)** Overview of the proposed semi-supervised segmentation approach. We utilize an unsupervised loss to obtain a better representation for the radiographs and a supervised loss to relate those features to the segmentation task. **(B)** Overview of the proposed unsupervised domain adaptation (UDA) segmentation approach. We leverage U-shaped GAN to find a better representation with basic features supporting radiograph imaging with an unsupervised loss. The distributions of the source and target data are aligned in this representation.

Under this hypothesis, we use the unannotated and annotated data to find a representation for the radiographs. A particular semi-supervised loss, which can be divided into a supervised loss and an unsupervised loss, is proposed. The supervised loss using the annotated data is employed for segmentation prediction, and the unsupervised loss using the unannotated data is utilized for a better representation of the whole dataset, as shown in Figure 1A. The unannotated data generalize the model as a regularizer. In U-shaped GAN, we employ a generator to generate realistic data segmented by a multiclass classifier (our discriminator) from the noise input, which in addition to classifying the pixels into lungs, determines whether a given pixel belongs to the real or generated data. The generator converges the realistic data to the real distribution *p*(*x*) of the partly annotated real data. This enables the discriminator to learn better features to represent the radiographs and to filter irrelevant individual features. Moreover, we employ the annotated data to find the relations among those features and the segmentation task. We modify a GAN by replacing the original discriminator with a U-shaped net (24) for assigning a label to each pixel. Training convolutional neural networks (CNNs) with a high resolution is effective for lung segmentation (20, 21). U-shaped GAN is designed for high-resolution chest radiographs. Unlike the segmentation GAN (1), we use the semantic classes and the background as an additional class for segmentation as the background contains several unannotated organs that reflect the imaging condition.

**Figure 2:**
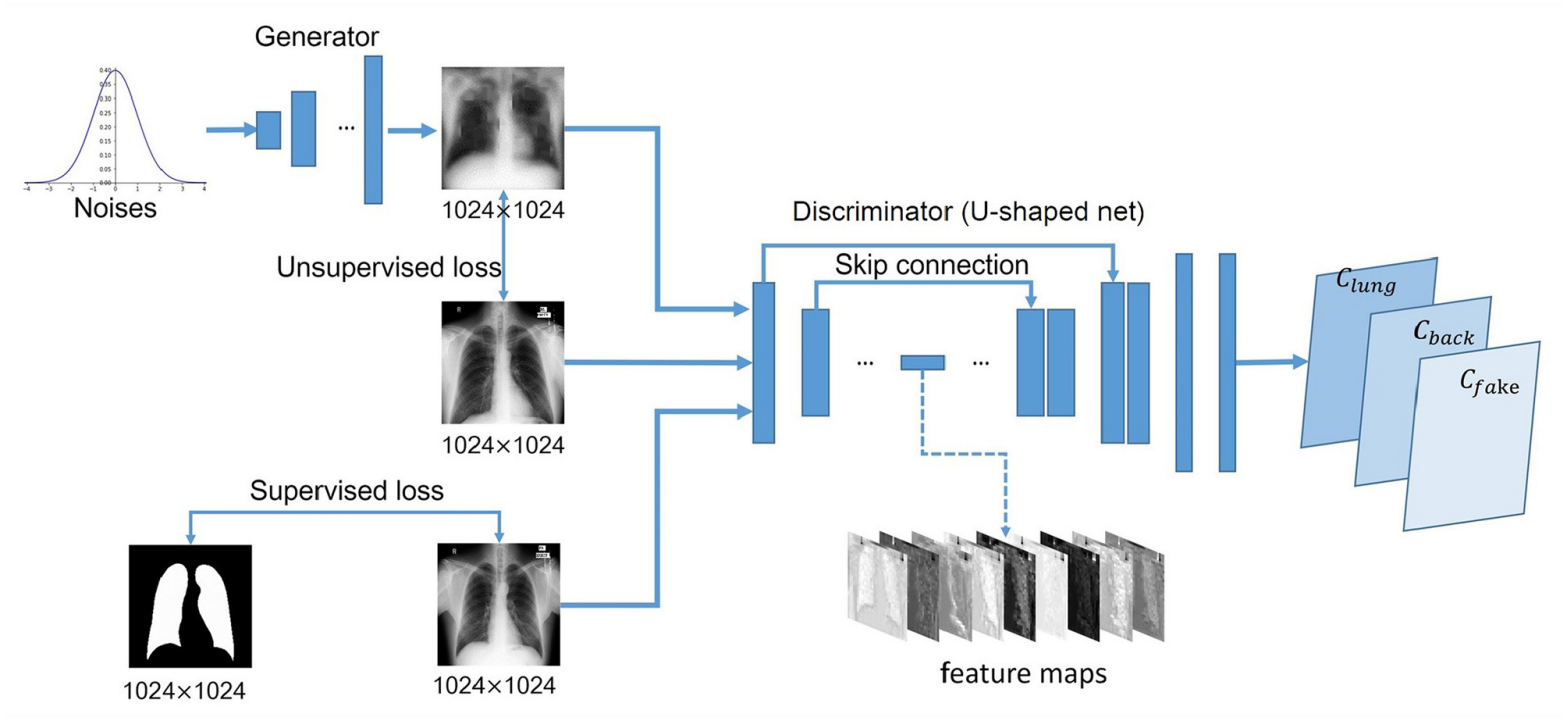
Schematic description of U-shaped GAN. The generator transforms noise into fake images. The fake and real data are used to train the discriminator for pixel-level predictions as lungs *C*_*lung*_, background *C*_*back*_, and fake *C*_*fake*_.

We improve GAN by replacing the traditional discriminator with a U-shaped net, which, instead of predicting each image a label, assigns to each pixel a label. The proposed end-to-end deep learning model is illustrated in Figure 2. The discriminator acts as a segmentation network to assign one of the following labels to each pixel: lung class, background, or fake data. The annotated data is used to train the discriminator *D* to minimize the loss function *L*_*l*_:


(3) $$\begin{array}{l} \begin{array}{c} L_{l}=-E_{x\sim p_{data_{l}}\left(x,y\right)}\left[\log\left(D\left(y|x\right)\right)\right], \end{array} \end{array}$$

where *p*_*data*_*l*__(*x, y*) is the joint distribution of the pixel-level labels *y* and pixel values *x* of the annotated data; the discriminator *D* predicts the possibility *D*(*y*|*x*) of pixel *x* belonging to label *y*.

In semi-supervised learning, where the labels are partly available among the training images, it is convenient to leverage the unannotated data for estimating a representation with useful features for segmentation. The true labels *y* of the pixels of the unannotated data are set as real data. The loss function for training the discriminator *D* with the unannotated data is defined as follows:


(4) $$\begin{array}{l} \begin{array}{l} L_{u}=-E_{x\sim p_{data_{u}}\left(x\right)}\left[\log\left(D\left(y|x\right)\right)\right]\\ \;=-E_{x\sim p_{data_{u}}\left(x\right)}\left[\log\left(1-D{\left(x\right)}_{fake}\right)\right], \end{array} \end{array}$$

where *p*_*data*_*u*__(*x*) is the distribution of pixels of chest radiographs without annotation; *D*(*x*)_*fake*_ is the possibility of the pixel belonging to the fake data. In U-shaped GAN, there is no output designed with the label “real data.” We used the 1 − *D*(*x*)_*fake*_ instead.

The generator *G* maps a random noise *z* to a sample *G*(*z*) that is close to chest radiographs, while the discriminator *D* is trained to label the generated sample *G*(*z*) as fake. The true labels *y* of the pixels of the generated data are set as fake data. The loss function for the discriminator *D* with the generated data is given as


(5) $$\begin{array}{l} \begin{array}{c} L_{g}=-E_{z\sim p_{noise}\left(z\right)}\left[\log\left(D{\left(G\left(z\right)\right)}_{fake}\right)\right], \end{array} \end{array}$$

where *D*(*G*(*z*))_*fake*_ is the possibility of the generated pixel belonging to the fake data.

We minimize the pixel-level discriminator loss *L*_*D*_ with respect to three types of input data, as follows:


(6) $$\begin{array}{l} \begin{array}{c} L_{D}=-E_{x\sim p_{data_{u}}\left(x\right)}\left[\log\left(1-D{\left(x\right)}_{fake}\right)\right]\\ \;-E_{z\sim p_{noise}\left(z\right)}\left[\log\left(D{\left(G\left(z\right)\right)}_{fake}\right)\right]\\ \;-E_{x\sim p_{data_{l}}\left(x,y\right)}\left[\log\left(D\left(y|x\right)\right)\right]. \end{array} \end{array}$$

The first and second terms of *L*_*D*_ are devised for unannotated data as an unsupervised loss to increase the ability of the discriminator to identify the real radiographs from fake ones and to find salient features in the chest radiograph. The third term is devised for annotated data as a supervised loss training *D* to find correct relations among these features and the segmentation task. We use a soft maximum over the outputs. *D*(*y*|*x*) for the annotated data is a component of the 1−*D*(*x*)_*fake*_. Increasing the probability *D*(*y*|*x*) will decrease the probability of *D*(*x*)_*fake*_. The third term has the same effect as the first term and acts as an unsupervised loss to increase the ability of the discriminating and salient features finding. We minimize the generator loss *L*_*G*_ to train the generator *G* as follows:


(7) $$\begin{array}{l} \begin{array}{c} L_{G}=E_{z\sim p_{noise}\left(z\right)}\left[\log\left(D{\left(G\left(z\right)\right)}_{fake}\right)\right]. \end{array} \end{array}$$

Because all of the annotated and unannotated data contribute to the discriminating ability of U-shaped GAN, *G* generates a distribution *p*_*g*_(*G*(*z*)) converging to the real distribution *p*(*x*) of the whole dataset consisting of annotated and unannotated radiographs.

#### 2.2.2. UDA Approach

The chest radiographs from various sources acquired by the same imaging modality differ in three aspects: image quality, image appearance, and spatial configuration (8). The features relevant to the basic imaging causes among the radiographs are similar. Based on this property, we aim to develop a UDA approach to find these similar features, filter features of individual domains, and align the source and target domains in a representation, which is similar to our semi-supervised learning approach. U-shaped GAN is extended to UDA with minimal modification and uses nearly the same training process in the semi-supervised learning and UDA approaches. We use a U-shaped GAN to search for the aforementioned features with the annotated source domain and unannotated target domain, as shown in Figure 1B. The target data serves as the unannotated data in the semi-supervised learning approach to generalize the model trained on the source dataset. The source data serves as the annotated data to ensure the accuracy of the segmentation task.

U-shaped GAN is extended to UDA, where only the source data are annotated, and the labels of target data are not available. The source and target domain data are used as the annotated and unannotated data in *L*_*D*_, respectively, to train the discriminator. The generator loss function is the same as *L*_*G*_ in the semi-supervised approach. The generator *G* produces the fake data matching the aligned representation of the source and target data in the min-max game of *G* and *D*. The fake data are close to the real radiographs in both domains, and the discriminator learns better features related to the basic imaging causes of radiographs. U-shaped GAN then finds a suitable representation for the radiographs of the target and source data. The distributions of source and target data are aligned in this representation. *L*_*D*_ and *L*_*G*_ encourage domain-invariant detectors to emerge in U-shaped GAN. The annotated source data also guarantees the correct segmentation prediction.

### 2.3. Network Architecture

**Figure 3:**
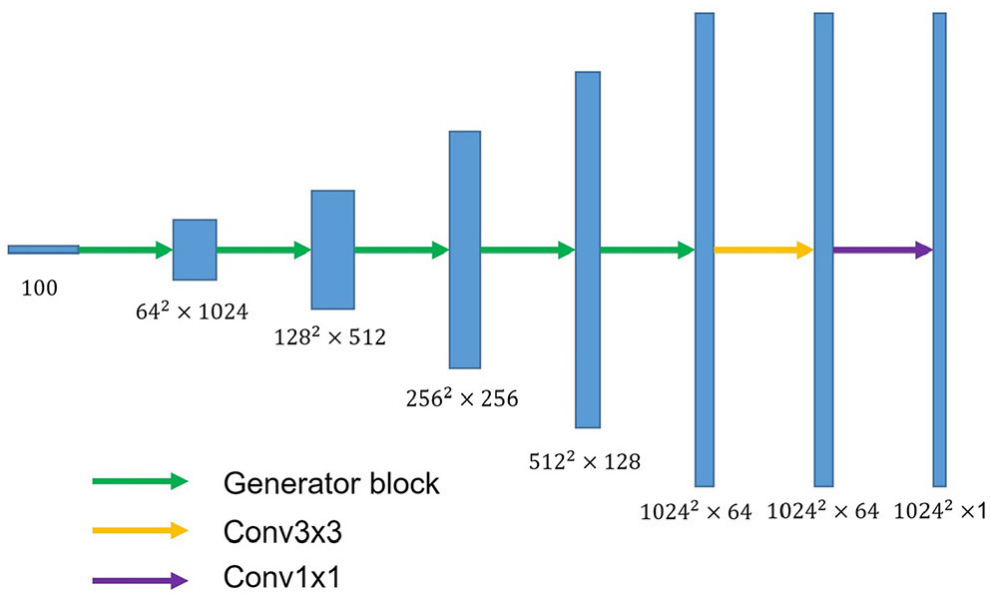
The overall structure of the proposed generator. The generator block consists of a pointwise convolution layer and two 4 × 4 fractionally strided convolution layers. Conv3×3 denotes the 3 × 3 convolution layer. Conv1×1 represents the 1 × 1 convolution layer.

**Figure 4:**
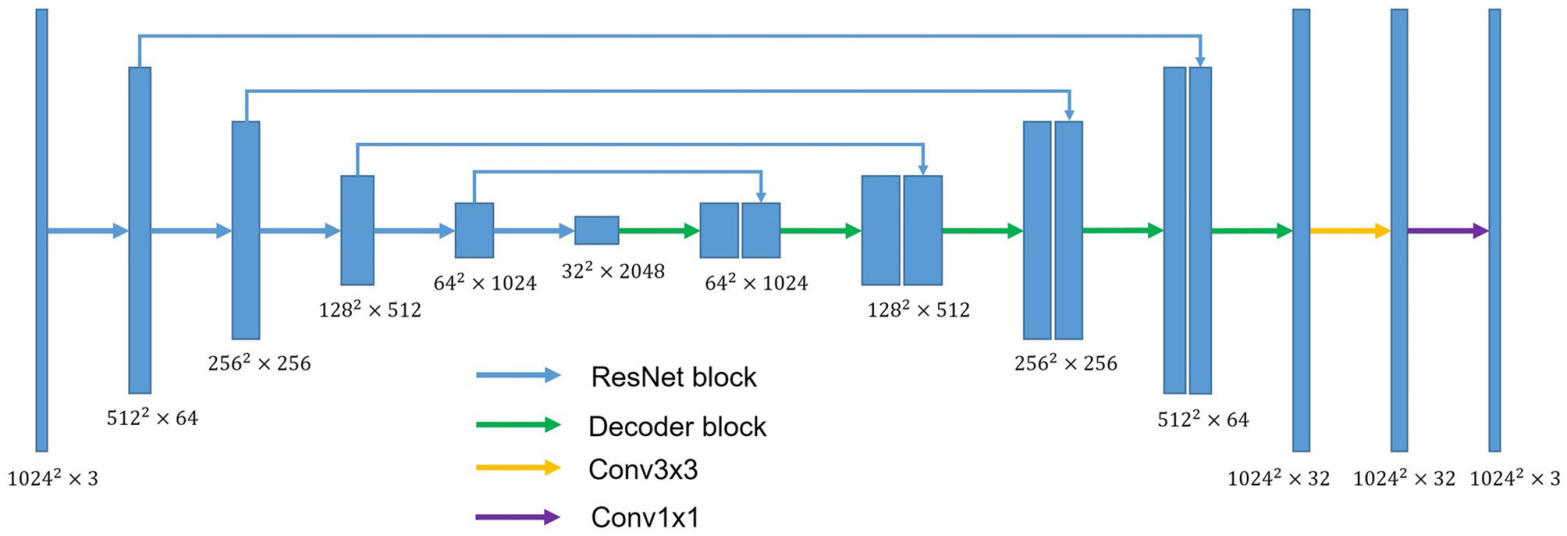
The overall structure of the discriminator. ResNet block stands for the block used in ResNet-50. Conv3×3 indicates the 3 × 3 convolution layer. Conv1×1 indicates the 1 × 1 convolution layer. Decoder block mainly comprises a 3 × 3 convolution layer and 4 × 4 fractionally strided convolution layer. A pointwise convolution layer is used to connect them.

U-shaped GAN is proposed to label each input image pixel *y* as a lung, background, or fake pixel. The U-shaped net is incorporated into the structure of GAN, serving as the discriminator, to label each pixel. A schematic description of U-shaped GAN is shown in Figure 2. Training CNNs at a high resolution is effective for lung segmentation predictions; however, it increases the computational burden (20, 21). Therefore, we use a pointwise convolution layer followed by two 4 × 4 fractionally strided convolution layers to form a new generator block (Figure 3), instead of the fractionally strided convolution layers used in deep convolutional GANs (25), to achieve a high resolution of 1, 024 × 1, 024. The pointwise convolution layer reduces feature dimensions while retaining the salient features and leaves the 4 × 4 layers with fewer parameters. This approach significantly reduces the computational complexity. The model parameters decrease from 1.34 × 10^8^ to 1.35 × 10^7^ and the floating-point operations (FLOPs) decrease from 5.84 × 10^8^ to 1.53 × 10^8^. In addition, we improve U-shaped GAN with a modified U-shaped network as the discriminator, as shown in Figure 4. The U-shaped net consists of a feature encoder and decoder modules (24). We replace the encoder with a pretrained ResNet-50 (26), which further reduces the computational burden of training from scratch. Moreover, ResNet-50 solves the degradation problem by adding identity connections to the convolution network (26). The feature decoder module restores the high-level semantic features extracted from the feature encoder module. The modified decoder module comprises four building blocks, as shown in Figure 4. The fundamental building block mainly comprises a 3 × 3 convolution layer followed by a 4 × 4 fractionally strided convolution layer. A pointwise convolution layer is used to connect them to reduce relevant parameters. Skip connections take information directly from the encoder to the decoder layers and recover the information loss due to consecutive pooling and striding convolutional operations (24). We use instance normalization (27) followed by LeakyReLU activation functions (28) between each layer.

## 3. Experiments

### 3.1. Datasets

In our experiments, we utilize the Japanese Society of Radiological Technology (JSRT) (29) and Montgomery County (MC) datasets (30, 31). The JSRT dataset contains 247 posterior-anterior (PA) chest radiographs, of which 154 contain lung nodules and 93 have no nodules (29). The ground truth lung masks can be obtained in the Segmentation in Chest Radiographs dataset (32). The MC dataset contains PA chest radiographs collected from the National Library of Medicine, National Institutes of Health, Bethesda, MD, USA. It consists of 80 normal and 58 abnormal cases with manifestations of tuberculosis (30, 31). The ground truth lung masks are also contained in the MC dataset.

### 3.2. Metrics

Several algorithms with different evaluation metrics are available in the literature. We used two commonly used methods, the Jaccard index and Dice score metrics, to compare U-shaped GAN with the state-of-the-art models.

(1) The Jaccard index statistic is used for gauging the similarity and diversity of sample sets. It shows the agreement between the ground truth B and the predicted set of pixels A and is given as:


(8) $$\begin{array}{l} J\left(A,B\right)=\frac{\left|A\cap B\right|}{\left|A\cup B\right|}. \end{array}$$

(2) The Dice score measures the overlap between the ground truth B and the predicted set of pixels A as follows:


(9) $$\begin{array}{l} D\left(A,B\right)=\frac{2\times |A\cap B|}{\left|A|+|B\right|}. \end{array}$$

### 3.3. Implementation Details

U-shaped GAN is implemented in Python using the PyTorch framework. The gray chest radiographs are resized to 1, 024 × 1, 024 and converted to RGB images compatible with the pre-trained ResNet-50 before placement in the network. The weights of ResNet-50 in our discriminator are pre-trained on the ImageNet dataset (33). The default 5-fold cross-validation is considered to train the semi-supervised model. In the semi-supervised approach, U-shaped GAN is trained with the JSRT or MC dataset with a portion of pixel-wise annotated data and the remainder without pixel-level annotations with 500 epochs. The Adam optimizer (34) is used to train 500 epochs of the generator and discriminator with initial learning rates of 0.001 and 0.0001, respectively, and multiplied by 0.1 after 200 epochs. For semi-supervised learning, we randomly select 12.5, 25, and 50% of the radiographs from the training set as the annotated set, with the remainder forming the unannotated set. Moreover, we train our model with 100% of the training dataset in the supervised approach. An ablation study is performed to discover the performance contribution from the modified architecture of U-shaped GAN and the proposed pixel-level semi-supervised loss. As in Li et al. (35), we train U-shaped GAN *via* the semi-supervised approach and supervised approach with 35 annotated radiographs on the JSRT dataset and with 24 annotated radiographs on the MC dataset. The supervised approach is conducted solely with the same annotated set and segmentation network. Moreover, we explore the effect of U-shaped GAN with the original GAN loss, called the original approach, by adding classification layers to U-shaped GAN paralleling with the decoder of our discriminator. The classification layers are identical to those in ResNet-50. In the original approach, the classification layers discriminate the real radiographs from the fake ones, and our discriminator just works as a segmentation network to predict the probability of belonging to the lungs of each pixel in the original approach. For UDA, we first employ the MC and JSRT datasets as the source and target domains, respectively, and then swap their roles. We randomly split each dataset into 7:1:2 for training, validation, and test sets. We train U-shaped GAN similarly to the semi-supervised approach using the source and target data as the annotated and unannotated data, respectively.

## 4. Results

### 4.1. Semi-Supervised Segmentation

U-shaped GAN is trained on the MC and JSRT datasets independently. The comparison with the state-of-the-art semi-supervised CNN (35) is shown in Table 1. As few semi-supervised models on chest radiographs are available, we also compare U-shaped GAN with 1) human observation (32); 2) traditional methods (30, 36); and 3) supervised CNNs (21, 35, 37, 38). The comparison is shown in Table 2.

**Table 1:** Comparison of U-shaped generative adversarial network (GAN) with the state-of-the-art semi-supervised model.

**Dataset**	**Model**	**Main method**	**Dice**	**IoU**
JSRT	Li (35)	Semi-supervised CNN(35)	0.967	-
	U-shaped GAN	Semi-supervised GAN(35)	**0.971**	**0.944**

*The numbers after the main method are the numbers of the annotated radiographs used in the semi-supervised approach. Numbers in bold indicate the best result among the models*.

**Table 2:** Comparison of U-shaped GAN with other lung segmentation methods for chest radiograph datasets.

**Dataset**	**Model**	**Main method**	**Dice**	**IoU**
JSRT	Human (32)	Human observation	-	0.946 ± 0.018
	Candemir (30)	Traditional method	0.967 ± 0.008	0.954 ± 0.015
	U-net (35)	Supervised CNN	0.946	-
	InvertedNet (21)	Supervised CNN	0.974	0.950
	Li (35)	Supervised CNN	0.967	-
	U-shaped GAN	Supervised GAN	**0.979** **±** **0.001**	**0.958** ± **0.003**
	U-shaped GAN	Semi-supervised GAN(25%)	0.975 ± 0.001	0.951 ± 0.002
MC	Bosdelekidis (36)	Traditional method	0.923	0.862
	Candemir (30)	Traditional method	0.960 ± 0.018	0.941 ± 0.034
	U-net (37)	Supervised CNN	-	0.942 ± 0.046
	Souza (38)	Supervised CNN	0.936	0.881
	U-shaped GAN	Supervised GAN	**0.976** **±** **0.006**	**0.955** **±** **0.010**
	U-shaped GAN	Semi-supervised GAN(25%)	0.968 ± 0.011	0.940 ± 0.019

*The number after the main method is the proportion of annotated radiographs from the training set in the semi-supervised approach. Numbers in bold indicate the best result among the models*.

U-shaped GAN trained with 100% annotated data achieves a performance increase of 0.4–10.8% over the state-of-the-art traditional models and supervised CNNs on both the JSRT and MC datasets. The results validate the effectiveness of the design of the segmentation network.

**Figure 5:**
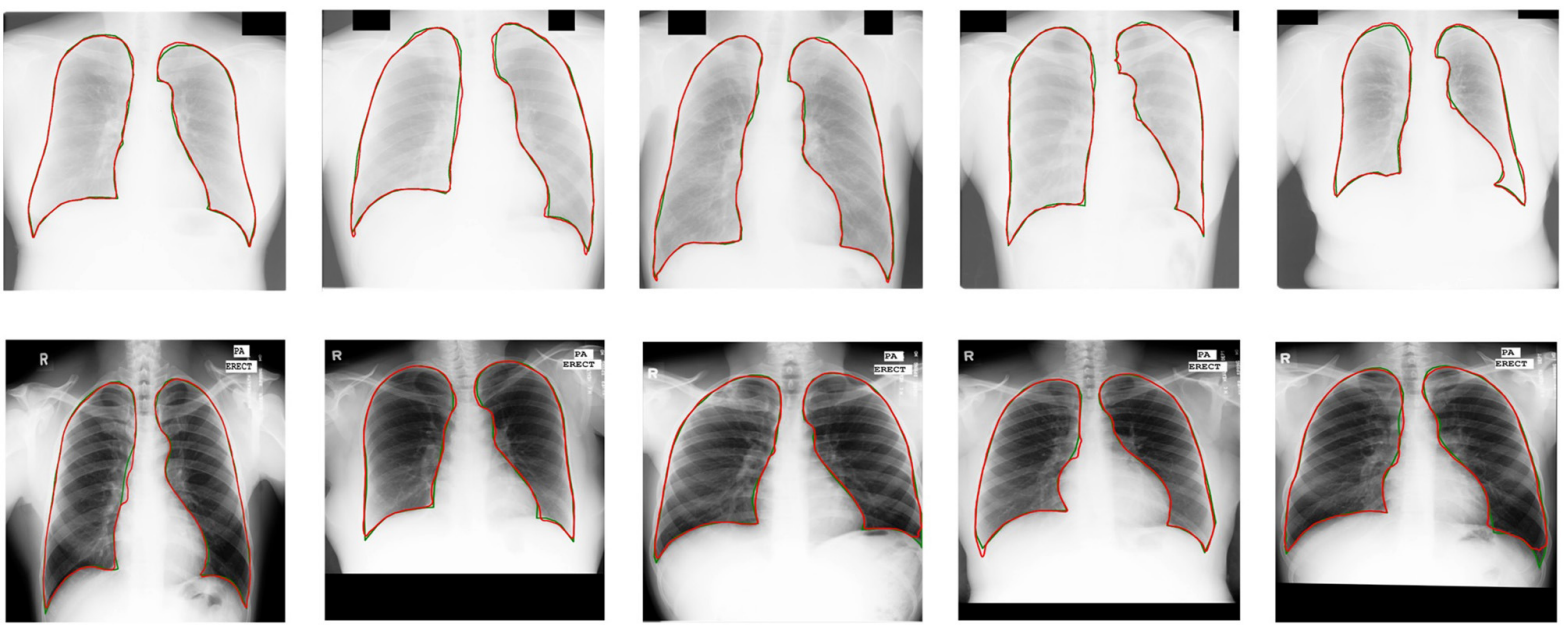
Semi-supervised results (25% annotated data) with U-shaped GAN. The radiographs from the Japanese Society of Radiological Technology (JSRT) dataset and Montgomery County (MC) dataset appear at the top and bottom, respectively. Green and red contours indicate the true ground and automatic segmentation results, respectively.

Our semi-supervised model (Dice = 0.975, IoU = 0.951) trained with 25% annotated data outperforms the state-of-the-art supervised models and human observation on the JSRT dataset. Our semi-supervised model (Dice = 0.968, IoU = 0.940) trained with 25% annotated data outperforms the most state-of-the-art supervised models but performs slightly worse than the U-net model (IoU = 0.942) on the MC dataset. Our proposed semi-supervised model achieves outstanding performance with limited annotated datasets. Moreover, U-shaped GAN outperforms the state-of-the-art semi-supervised model (35) in both supervised and semi-supervised settings by 1.2 and 0.8%, respectively. Figure 5 shows a few examples of semi-supervised results with U-shaped GAN. The ground truth contour of the lungs is shown in green, and the segmentation result of the algorithm is in red.

We evaluate our approach with 12.5, 25, 50, and 100% annotated radiographs (remaining portions consist of unannotated radiographs). The annotated radiographs in the data splits are randomly sampled from the whole dataset. Notably, the approach works well even with 12.5% annotated data, as shown in Table 3. For the details, readers are referred to Supplementary Figures 1, 2.

**Table 3:** Comparison of the results of U-shaped GAN trained with different proportions of annotated data.

**Dataset**	**Annotated data (%)**	**Dice**	**IoU**
JSRT	100	0.979 ± 0.001	0.958 ± 0.003
	50	0.977 ± 0.002	0.956 ± 0.003
	25	0.975 ± 0.001	0.951 ± 0.002
	12.5	0.964 ± 0.007	0.934 ± 0.008
MC	100	0.976 ± 0.006	0.955 ± 0.010
	50	0.973 ± 0.007	0.949 ± 0.013
	25	0.968 ± 0.011	0.940 ± 0.019
	12.5	0.958 ± 0.021	0.922 ± 0.034

We apply U-shaped GAN on a pneumothorax segmentation dataset (39) with a semi-supervised approach. This dataset contains 2,669 radiographs with annotated pneumothorax lesion areas. U-shaped GAN shows promising performance on the pneumothorax segmentation in the semi-supervised approach. Most of the results predict rough areas of the pneumothorax lesion correctly, which provides credible help to the radiologist to find the lesion rapidly. For the details, readers are referred to Supplementary Figure 3.

### 4.2. Unsupervised Domain Adaptation

We use the MC and JSRT datasets as the source and target domains, respectively, and then swap their roles for UDA. The performances of our UDA model on the target domains are compared under various settings: 1) the model being trained on source data and tested on the target domain with no DA (T-noDA); 2) UDA model testing on the source domain (S-test); 3) human observation (32); 4) UDA models with CNNs (15, 37).

As shown in Table 4, when directly applying the learned source domain model to target data, the model performance significantly degrades, indicating that domain shift would severely impede the generalization performance of CNNs. However, remarkable improvements are achieved by applying the unsupervised loss on the target images. Compared to the T-noDA results, the segmentation predictions increase by 3.3% and 5.3% on the JSRT and MC datasets, respectively, with our UDA approach.

**Table 4:** Comparison of segmentation results among different unsupervised domain adaptation (UDA) methods.

**Dataset**	**Model**	**Main method**	**Dice**	**IoU**
MC → JSRT	Human observation (32)	Human observation	-	0.946
	T-noDAg	-	0.934	0.895
	S-test	-	0.981	0.963
	MUNIT (37)	UDA with MUNIT	-	0.882
	CyUDA (15)	UDA with CycleGAN	0.928	-
	SeUDA (15)	UDA with CycleGAN	0.945	-
	Our method	UDA with GAN	**0.965**	**0.932**
JSRT → MC	T-noDAg	-	0.918	0.880
	S-test	-	0.980	0.961
	Our method	UDA with GAN	**0.967**	**0.936**

*Numbers in bold indicate the best result among the models*.

**Figure 6:**
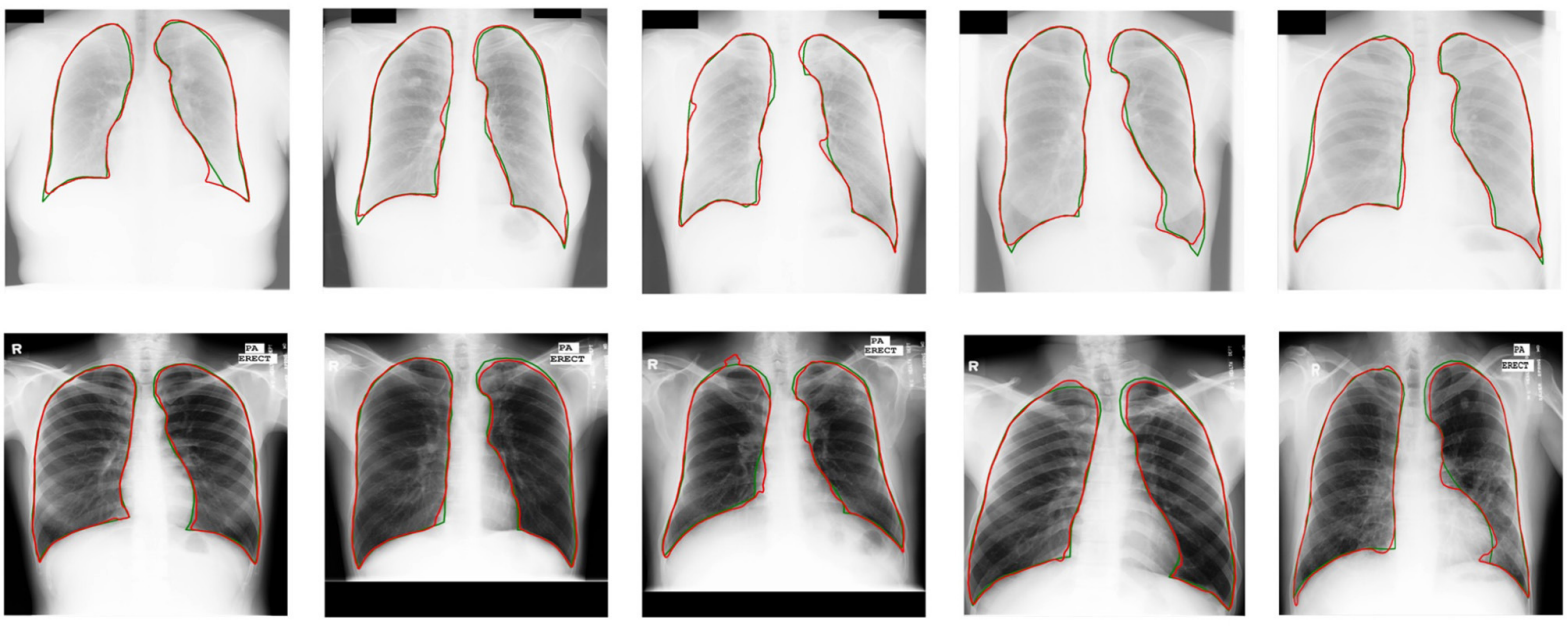
UDA results with U-shaped GAN. The radiographs from the JSRT dataset with MC → JSRT UDA results are on the top; the radiographs from the MC dataset with JSRT → MC UDA results are on the bottom. Green and red contours indicate the ground truth and automatic segmentation results, respectively.

Experimental results demonstrate a significant enhancement in performance compared to other models. Compared with other UDA models based on CNNs (15, 37), U-shaped GAN achieves a significant improvement over MUNIT (5.7%), CyUDA (4.0%), and SeUDA (2.1%). Moreover, U-shaped GAN is even comparable to human observation. Figure 6 shows a few examples of UDA results with U-shaped GAN. The ground truth contour of the lungs is shown in green, and the segmentation result of the algorithm is in red.

Compared to models trained with 100% annotated data on source domains, the UDA models obtain similar accuracy on the source domains (*S*-test). Therefore, U-shaped GAN is able to improve results on the target domains while maintaining segmentation performance on the source domains. For the details, readers are referred to Supplementary Figures 4, 5.

### 4.3. Ablation Study

For the ablation study, we study the effectiveness of our modified segmentation network, the architecture of U-shaped GAN, and the proposed pixel-level semi-supervised loss. To demonstrate the advantage of U-shaped GAN, we report the scores of U-net (the original U-shaped net), U-shaped GAN trained with the supervised approach, U-shaped GAN trained with the original approach, and U-shaped GAN trained with our pixel-level loss.

First, the results of the comparison of our modified segmentation network and the original U-shaped net are shown in Table 5. The segmentation network of U-shaped GAN is designed with high resolution radiographs following the main idea of the U-shaped net (24). It is shown that our modified segmentation network improves the prediction by 3.28% on the JSRT dataset and 2.02% on the MC dataset when trained with the whole annotated datasets.

**Table 5:** Comparison of U-shaped GAN and U-net trained with the whole dataset.

**Dataset**	**Model**	**Main method**	**Dice**	**IoU**
JSRT	U-net (35)	Supervised approach	0.946	-
	U-shaped GAN	Supervised approach	**0.977**	**0.955**
MC	U-net (37)	Supervised approach	-	0.942
	U-shaped GAN	Supervised approach	**0.980**	**0.961**

*Numbers in bold indicate the best result among the models*.

Second, the effectiveness of the architecture of U-shaped GAN is investigated. The U-shaped net is incorporated into the structure of GAN leveraging unannotated data to assist the segmentation task. By adding an original GAN loss to the supervised approach, the Dice scores increase from 0.968 to 0.970 on the JSRT dataset and from 0.966 to 0.971 on the MC dataset. The architecture of U-shaped GAN is successful in leveraging unannotated data to find a representation for the whole dataset, shown in Table 6.

**Table 6:** Comparison of U-shaped GAN with different training approaches.

**Dataset**	**Model**	**Main method**	**Dice**	**IoU**
JSRT	U-shaped GAN	Supervised approach(35)	0.968	0.939
	U-shaped GAN	Original approach(35)	0.970	0.941
	U-shaped GAN	Semi-supervised approach(35)	**0.971**	**0.944**
MC	U-shaped GAN	Supervised approach(24)	0.966	0.936
	U-shaped GAN	Original approach(24)	0.971	0.945
	U-shaped GAN	Semi-supervised approach(24)	**0.973**	**0.948**

*The number after the main method is the number of the annotated radiographs used in training. Numbers in bold indicate the best result among the models*.

Third, we show the comparison between results gained by the GAN original loss and our pixel-level GAN loss with semi-supervised training. The pixel-level GAN loss increases the capacity of U-shaped GAN in finding the representation of the whole dataset. The segmentation results improve from Dice = 0.970, IoU = 0.941 to Dice = 0.971, IoU = 0.944 on the JSRT dataset and Dice = 0.971, IoU = 0.945 to Dice = 0.973, IoU = 0.948 on the MC dataset, shown in Table 6.

**Figure 7:**
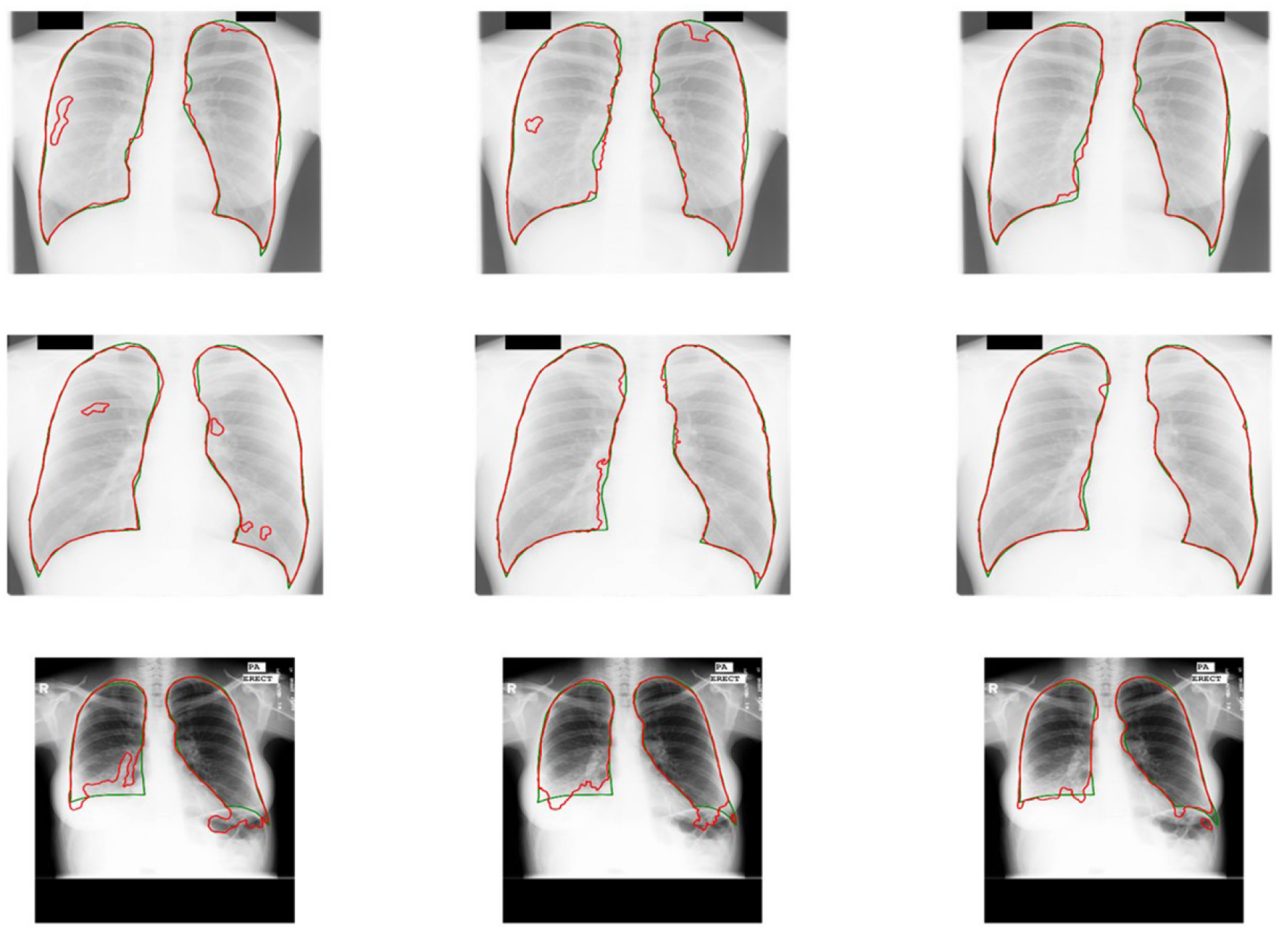
Comparison of the results of U-shaped GAN with different training approaches on confusing samples. The radiographs in the first row are results of the supervised approach. The second row is the results of the original approach. The third row is the results of the semi-supervised approach. Green and red contours indicate the ground truth and automatic segmentation results, respectively.

Some results of U-shaped GAN with different training approaches on confusing samples are shown in Figure 7. U-shaped GAN seems to be resistant to interference by irrelevant features, such as other organs and lesion areas, by using the GAN architecture and to increase the resistant capability by the pixel-level loss.

## 5. Discussion

In this study, we propose U-shaped GAN to address the scarcity of annotated data and domain shift. U-shaped GAN for radiographs shows strong performance in semi-supervised learning and UDA approaches. To handle datasets from multiple medical centers conveniently and efficiently, we combine semi-supervised learning and UDA in radiograph segmentation into a single method. U-shaped GAN functions similarly with the annotated and unannotated data in the semi-supervised and UDA approaches. The effectiveness of the model is demonstrated through extensive experiments.

Training models with high image resolution is effective; however, it would increase the computational burden (20, 21). The previous models concentrated on the 256 × 256 or 512 × 512 image resolutions (15, 21, 35, 37, 38). We propose U-shaped GAN for high resolution radiographs (1, 024 × 1, 024). We use pointwise convolution for dimensionality reduction, decreasing the number of feature maps while retaining their salient features. Moreover, we design the U-shaped net with a pretrained ResNet-50 as encoder, which further reduces the computational burden of the training encoder from scratch. U-shaped GAN trained with the whole annotated data achieves a more accurate performance than the state-of-the-art supervised models as well as the original U-shaped net. This prediction result shows that U-shaped GAN is effective for segmentation prediction.

The previous studies often analyzed semi-supervised learning and UDA problems separately (3, 11, 12, 14–16). In semi-supervised learning, the previous GANs were usually proposed to distinguish between segmentation probability maps and the ground truth (3, 11, 12). The generators produced the segmentation probability maps as the segmentation networks (3, 11, 12). In UDA, the previous GANs were usually proposed to distinguish between source data and target data (14–16). The generators transferred the target domain to the source domain and extra networks were designed for the segmentation (14–16). The annotated and unannotated data may come from either the same or different domains in the dataset collected from multiple centers. Dealing with the two problems separately increases the model complexity. Moreover, separating the dataset to train two segmentation networks decreases the utilization efficiency of collected data. Therefore, we propose a single model to deal with the semi-supervised learning and UDA approaches at the same time. We attribute the model's remarkable generalization capabilities to the effective use of the unannotated data. We use adversarial learning to achieve a representation for lung segmentation in chest radiographs. In U-shaped GAN, we employ a generator to generate realistic data, which, together with the real data (most of them are unannotated data), force the discriminator to find the most salient features. Our discriminator, which in addition to classifying the pixels into lungs, determines whether a given pixel belongs to the real or generated data.

U-shaped GAN exploits more widely available unannotated data to complement small annotated data with a semi-supervised loss. U-shaped GAN achieves greater performance than the state-of-the-art semi-supervised model. Moreover, it is comparable to the supervised models with 25% annotated data. U-shaped GAN works well even with 12.5% annotated data with Dice scores of 0.964 and 0.958 on JSRT and MC datasets, respectively. Unlike the previous semi-supervised study using the generators to produce the segmentation probability maps as the segmentation networks (3, 11, 12, 25), U-shaped GAN uses the generator to generate realistic data. The realistic data, together with the real data, force our discriminator to learn a better representation for the radiographs. Compared with the result achieved by trained in the supervised approach, U-shaped GAN achieves increased performance in the semi-supervised learning approach with adversarial learning. Instead of discriminating real or fake labels on image-level (3, 11, 12, 25), a pixel-level loss is proposed to extract more information from the radiographs. The segmentation accuracy is improved when using the proposed loss. For the data from one domain, U-shaped GAN effectively leverages the unannotated data to achieve high segmentation accuracy and reduces the cost of medical image annotation.

U-shaped GAN is extended to UDA to reduce domain shift without the extra expense of annotation on the target domain. Instead of transferring the target domain to the source domain (14–16, 37), our generator generates realistic data. Discriminating the realistic data from the real ones, our discriminator learns a better representation. U-shaped GAN is better than the state-of-the-art UDA models and comparable to human observation. It achieves high accuracy on the target domain while maintaining the accuracy on the source domain (S-test). Thus, the model can be trained with data collected from multiple medical centers. Regardless of whether the unannotated data come from single or multiple domains, the prediction accuracies on their corresponding domains are increased, and the accuracies on other domains are maintained. Because the same networks are used in the two approaches, the datasets are sufficient to train U-shaped GAN and generalize the model among various domains, making it suitable for clinical applications in a multiple center system. In addition, using the same architecture at multiple medical centers reduces the model complexity.

Results of our evaluation are promising, but U-shaped GAN has only been fully tested with lung segmentation. In the future, we will extend the model to detect a wider range of lung diseases by collecting additional chest radiographs of different diseases from multiple medical centers.

## 6. Conclusion

In this study, we propose U-shaped GAN to overcome the crucial problems caused by scarce labeled data and inevitable domain shift. The GAN-based model is designed at a high resolution (1, 024 × 1, 024) for effective segmentation. The semi-supervised learning approach and UDA approach are modeled into a unified framework for effective radiograph segmentation. We leverage unannotated and annotated data with a pixel-level semi-supervised loss. U-shaped GAN is compatible with varying data distributions of multiple medical centers, with efficient training and optimizing performance. Our experiment results demonstrate that U-shaped GAN achieved more accurate lung segmentation performance as compared with the state-of-the-art models. U-shaped GAN is more appealing to the model development and clinical application by eliminating the need to use two different models to deal with the aforementioned problems.

## Data Availability Statement

The original contributions presented in the study are included in the article/Supplementary Material, further inquiries can be directed to the corresponding author/s.

## Author Contributions

HG and HW conceived the idea for this study. HW worked on the end-to-end implementation of the study. JW provided relevant insights on the clinical impact of the research work and handled the redaction of the paper. PQ managed the project and provided the funding for the research. All authors contributed to the article and approved the submitted version.

## Funding

This work was supported by the National Natural Science Foundation of China (grant nos. 61633006 and 81872247) and the Fundamental Research Funds for the Central Universities, China (grant no. DUT21YG118).

## Conflict of Interest

The authors declare that the research was conducted in the absence of any commercial or financial relationships that could be construed as a potential conflict of interest.

## Publisher's Note

All claims expressed in this article are solely those of the authors and do not necessarily represent those of their affiliated organizations, or those of the publisher, the editors and the reviewers. Any product that may be evaluated in this article, or claim that may be made by its manufacturer, is not guaranteed or endorsed by the publisher.
