# Supplementary material for: U-shaped GAN for Semi-Supervised Learning and Unsupervised Domain Adaptation in High Resolution Chest Radiograph Segmentation
Source: Front Med (Lausanne). 2022 Jan 13;8:782664. doi: 10.3389/fmed.2021.782664 (PMC8792862; doi:10.3389/fmed.2021.782664)
Supplement: Supplementary file 1 [file Image_1.PDF]

Supplementary Material

1 SUPPLEMENTARY TABLES AND FIGURES

1.1 Figures

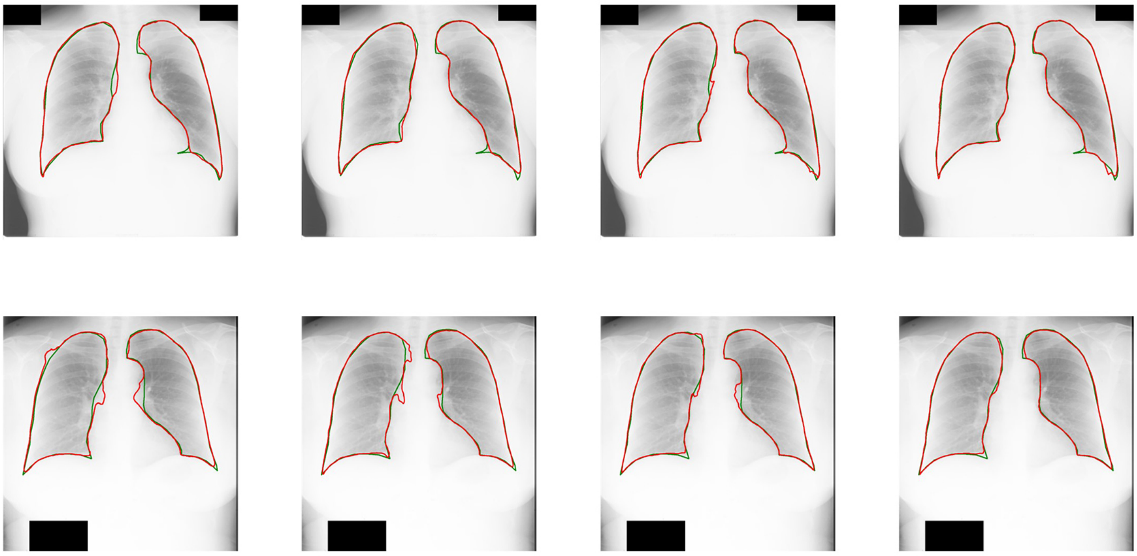

Figure S1. Comparison of the results of U-shaped GAN trained with different proportions on confusing samples on the JSRT dataset. The results in the first, second, third, and fourth row are trained with 12.5%, 25%, 50%, and 100%, respectively. Green and red contours indicate the ground truth and automatic segmentation results, respectively. The results on most of the samples are nearly the same, and only on several confusing samples, the segmentation errors occur at the edges of the lungs when the number of the annotated training data decreases.

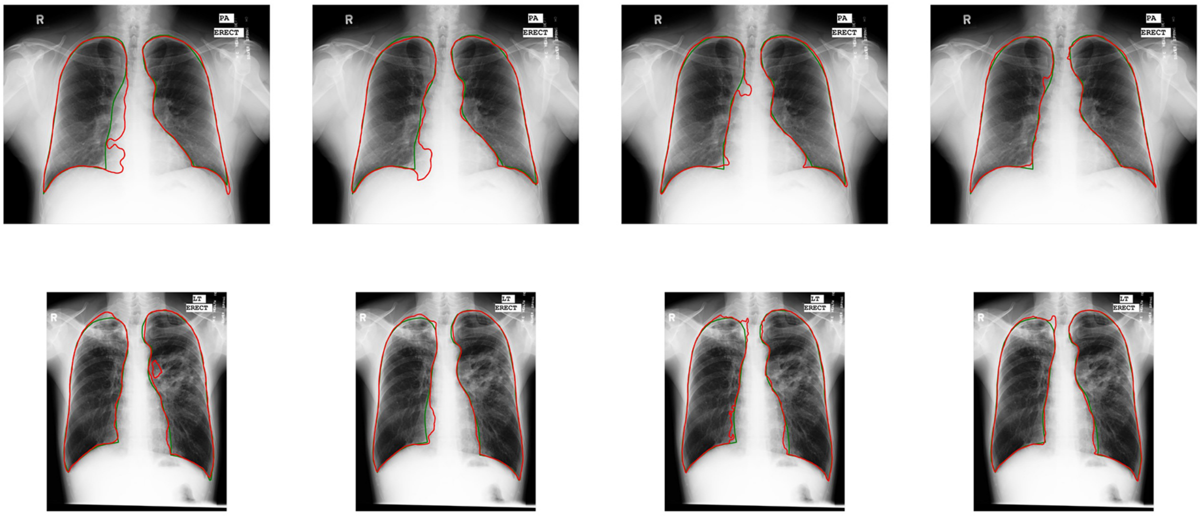

Figure S2. Comparison of the results of U-shaped GAN trained with different proportions on confusing samples on the MC dataset. The results in the first, second, third, and fourth row are trained with 12.5%, 25%, 50%, and 100%, respectively. Green and red contours indicate the ground truth and automatic segmentation results, respectively. The results on most of the samples are nearly the same, and only on several confusing samples, the segmentation errors occur at the edges of the lungs when the number of the annotated training data decreases.

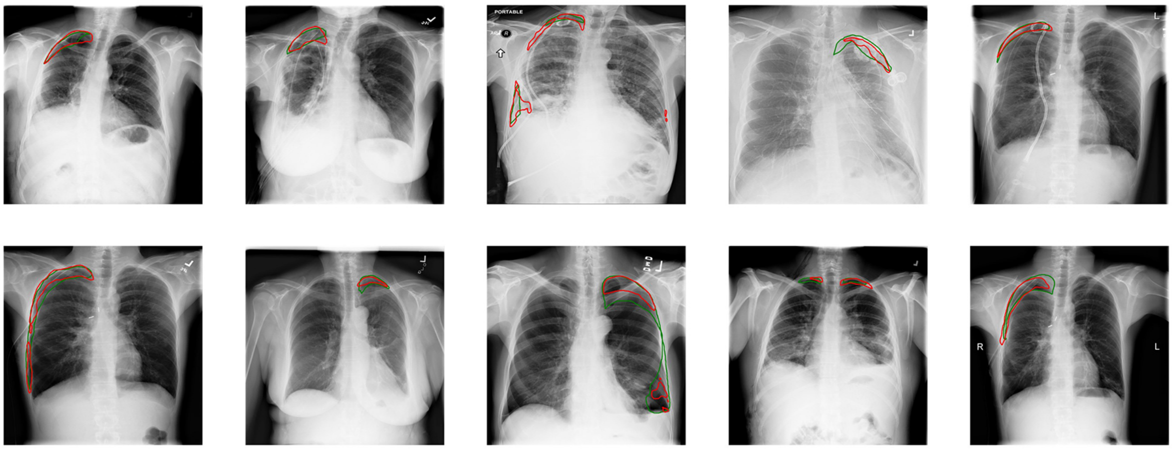

Figure S3. The segmentation results of U-shaped GAN on the pneumothorax with 25% annotated data. Green and red contours indicate the ground truth and automatic segmentation results, respectively. Most of the results can predict rough areas of the pneumothorax lesion correctly. This provides credible help to the radiologist to find the lesion rapidly.

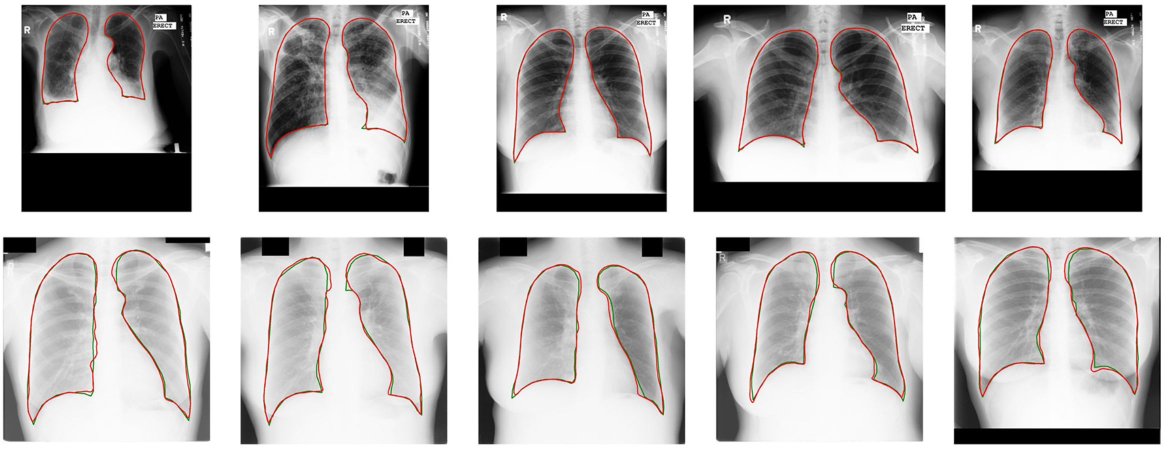

Figure S4. The results of source and target data with MC $\rightarrow$ JSRT UDA. U-shaped GAN improves results on the target domains while maintaining segmentation performance on the source domains. The results of the source dataset are on the top; The results of the target dataset are on the bottom. Green and red contours indicate the ground truth and automatic segmentation results, respectively.

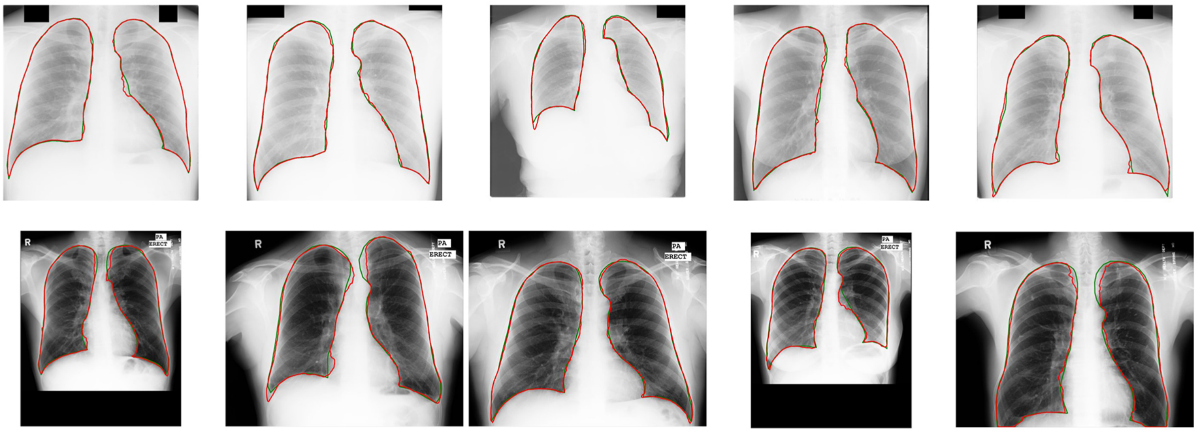

Figure S5. The results of source and target data with JSRT $\rightarrow$ MC UDA. U-shaped GAN improves results on the target domains while maintaining segmentation performance on the source domains. The results of the source dataset are on the top; The results of the target dataset are on the bottom. Green and red contours indicate the ground truth and automatic segmentation results, respectively.
